# Lactate regulates major zygotic genome activation by H3K18 lactylation in mammals

**DOI:** 10.1093/nsr/nwad295

**Published:** 2023-11-20

**Authors:** Jingyu Li, Weibo Hou, Qi Zhao, Wei Han, Hongdi Cui, Songling Xiao, Ling Zhu, Jiadan Qu, Xiaoyu Liu, Weitao Cong, Jingling Shen, Yuzheng Zhao, Shaorong Gao, Guoning Huang, Qingran Kong

**Affiliations:** Chongqing Key Laboratory of Human Embryo Engineering, Center for Reproductive Medicine, Chongqing Health Center for Women and Children, Women and Children's Hospital of Chongqing Medical University, Chongqing 400010, China; Oujiang Laboratory, Zhejiang Provincial Key Laboratory of Medical Genetics, Key Laboratory of Laboratory Medicine, Ministry of Education, School of Laboratory Medicine and Life Sciences, Wenzhou Medical University, Wenzhou 325035, China; Oujiang Laboratory, Zhejiang Provincial Key Laboratory of Medical Genetics, Key Laboratory of Laboratory Medicine, Ministry of Education, School of Laboratory Medicine and Life Sciences, Wenzhou Medical University, Wenzhou 325035, China; Chongqing Key Laboratory of Human Embryo Engineering, Center for Reproductive Medicine, Chongqing Health Center for Women and Children, Women and Children's Hospital of Chongqing Medical University, Chongqing 400010, China; Oujiang Laboratory, Zhejiang Provincial Key Laboratory of Medical Genetics, Key Laboratory of Laboratory Medicine, Ministry of Education, School of Laboratory Medicine and Life Sciences, Wenzhou Medical University, Wenzhou 325035, China; Oujiang Laboratory, Zhejiang Provincial Key Laboratory of Medical Genetics, Key Laboratory of Laboratory Medicine, Ministry of Education, School of Laboratory Medicine and Life Sciences, Wenzhou Medical University, Wenzhou 325035, China; Chongqing Key Laboratory of Human Embryo Engineering, Center for Reproductive Medicine, Chongqing Health Center for Women and Children, Women and Children's Hospital of Chongqing Medical University, Chongqing 400010, China; Chongqing Key Laboratory of Human Embryo Engineering, Center for Reproductive Medicine, Chongqing Health Center for Women and Children, Women and Children's Hospital of Chongqing Medical University, Chongqing 400010, China; Frontier Science Center for Stem Cell Research, School of Life Sciences and Technology, Tongji University, Shanghai 200092, China; School of Pharmaceutical Science, Wenzhou Medical University, Wenzhou 325035, China; Institute of Life Sciences, College of Life and Environmental Sciences, Wenzhou University, Wenzhou 325035, China; Optogenetics & Synthetic Biology Interdisciplinary Research Center, State Key Laboratory of Bioreactor Engineering, Shanghai Frontiers Science Center of Optogenetic Techniques for Cell Metabolism, School of Pharmacy, East China University of Science and Technology, Shanghai 2000237, China; Frontier Science Center for Stem Cell Research, School of Life Sciences and Technology, Tongji University, Shanghai 200092, China; Chongqing Key Laboratory of Human Embryo Engineering, Center for Reproductive Medicine, Chongqing Health Center for Women and Children, Women and Children's Hospital of Chongqing Medical University, Chongqing 400010, China; Oujiang Laboratory, Zhejiang Provincial Key Laboratory of Medical Genetics, Key Laboratory of Laboratory Medicine, Ministry of Education, School of Laboratory Medicine and Life Sciences, Wenzhou Medical University, Wenzhou 325035, China

**Keywords:** mammalian preimplantation development, lactate, epigenetic remodeling, histone lactylation, zygotic genome activation

## Abstract

Lactate is present at a high level in the microenvironment of mammalian preimplantation embryos *in vivo* and *in vitro*. However, its role in preimplantation development is unclear. Here, we report that lactate is highly enriched in the nuclei of early embryos when major zygotic genome activation (ZGA) occurs in humans and mice. The inhibition of its production and uptake results in developmental arrest at the 2-cell stage, major ZGA failure, and loss of lactate-derived H3K18lac, which could be rescued by the addition of Lac-CoA and recapitulated by overexpression of H3K18R mutation. By profiling the landscape of H3K18lac during mouse preimplantation development, we show that H3K18lac is enriched on the promoter regions of most major ZGA genes and correlates with their expressions. In humans, H3K18lac is also enriched in ZGA markers and temporally concomitant with their expressions. Taken together, we profile the landscapes of H3K18lac in mouse and human preimplantation embryos, and demonstrate the important role for H3K18lac in major ZGA, showing that a conserved metabolic mechanism underlies preimplantation development of mammalian embryos.

## INTRODUCTION

Mammalian preimplantation development, from zygote to blastocyst, involves a series of significant biological events, including zygotic gene activation (ZGA). ZGA is the first transcription event in life, which is characterized by activation of a group of genes (e.g. *Usp* genes). In mice, the initial ZGA occurs between the S phase of the zygote and G1 of the early 2-cell embryo, and is designated as minor ZGA to discriminate it from the burst of transcription that occurs during the mid-to-late 2-cell stage, which is designated as major ZGA [1–3]. Extensive epigenetic reprogramming plays an essential role in preimplantation embryo development in mammals, and is closely linked with ZGA. For example, the transition of broad H3K4me3 domains to transcriptional-start-site (TSS) regions is required for normal ZGA [4]. Thus, the regulation of epigenetic remodeling on ZGA needs to be further studied.

Cellular metabolism is the foundation of all biological activity, and its dynamics is directly linked to changes in epigenetics [5,6]. Metabolic reprogramming and its regulation are hardwired into the complex program of early embryo development in mammals, such as the role of reciprocal changes in a pair of competitive metabolites (e.g. α-KG and 2-HG) in dynamic erasure of histone methylation during early embryogenesis in mice [7]. We established a metabolome profile of preimplantation embryos of mice from the zygote to blastocyst stages, and found that the combination of the prominent metabolic cofactor NAD^+^ and the deacetylase SIRT1 removes zygotic H3K27ac in the late 2-cell stage for the exit of minor ZGA, and it is essential for mouse and human preimplantation embryo development [8]. Moreover, in the study, we observed that lactate showed high level in 2-cell embryos and close associations with the top 2-cell embryo genes, suggesting a functional role in ZGA.

Lactate, as a major nutrient, is at a high level in the developmental microenvironment both *in vivo* and *in vitro* and is readily oxidated as early as the zygote stage [9,10]. However, its role in preimplantation embryo development in mammals is unclear. Zhang *et al.* discovered a novel epigenetic modification, histone lactylation derived by lactate, that activates gene transcription [11]. In this study, we demonstrate that lactate is highly enriched in the nuclei of both human and mouse embryos when major ZGA occurs, and deprivation of lactate results in preimplantation development arrest at the 2-cell stage and major ZGA failure. Lactate-derived H3K18lac is enriched at the promoter regions of major ZGA genes and contributes to major ZGA. Our findings reveal an important role for H3K18lac in major ZGA of mammals, providing insight into the crosstalk between cell metabolism and epigenetic remodeling in the regulation of embryogenesis.

## RESULTS

### Lactate is highly enriched in the nuclei of 2-cell embryos and indispensable for preimplantation development in mice

**Figure 1.**
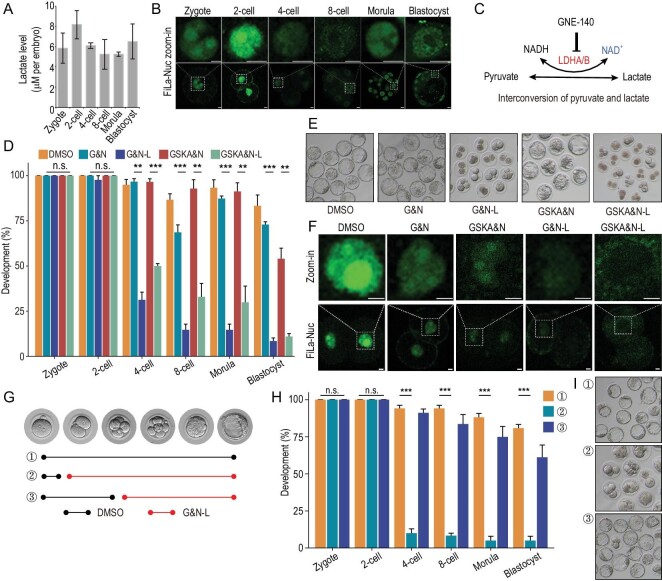
Lactate is essential for the preimplantation development of early embryos of mice. (A) Lactate levels during the development of preimplantation embryos in mice. Three experimental replicates were performed. Error bars represent SEM. (B) Fluorescence images of mouse embryos from different stages expressing FiLa in nucleus. Scale bars, 20 μm. (C) Interconversion of pyruvate and lactate. (D and E) Development rates of embryos in the groups. Three experimental replicates were performed. (F) Fluorescence images of mouse embryos at the 2-cell stage from different groups expressing FiLa in the nuclei at the 2-cell stage. Scale bars, 20 μm. (G) Overview of the experimental designs. (H and I) Development rates of embryos deprived of lactate at the late zygote and 4-cell stages. DMSO, mKSOM culture medium plus DMSO; G&N, mKSOM culture medium plus Gne140 and NMN; G&N-L, mKSOM culture medium without lactate plus Gne140 and NMN; GSKA&N, mKSOM culture medium plus GSK2837808A and NMN; GSKA&N-L, mKSOM culture medium without lactate plus GSK2837808A and NMN; Error bars are SEM. n.s. means not significant. **P < 0.01, ***P < 0.001. Differences between means were calculated using two-tailed Student's *t*-test.

We first measured lactate levels of preimplantation embryos of mice by absorptiometric analysis, and found the lactate level was not significantly high in 2-cell embryos (Fig. 1A). By using our recently reported lactate sensor [12], FiLa, we found no significant change in lactate level in the cytosol of embryos during preimplantation development (Fig. S1A). However, surprisingly, a remarkable lactate pool was observed in the nucleus of 2-cell embryos, comparing to cytosol, and also the lactate level in the nucleus of 2-cell embryos was higher than those at other stages (Fig. 1B), indicating that lactate in the nucleus may be important in early embryogenesis. However, the preimplantation development of embryos cultured in mKSOM lacking NaLac was not significantly affected (Fig. S1B). Pyruvate can be converted into lactate by lactate dehydrogenase (LDH; Fig. 1C), and both *Ldha* and *Ldhb* expressions were high in 2-cell embryos (Fig. S1C), and unexpectedly we found, through immunofluorescence (IF) staining, that LDH was highly enriched in the nucleus of 2-cell embryos (Fig. S1D), so we speculated that this conversion in the nucleus may compensate for the removal of lactate from the culture medium. Therefore, LDH activity was inhibited by Gne140 or GSK2837808A, dual LDHA/B inhibitors, and according to our previous study, nicotinamide mononucleotide (NMN) was added to rescue the loss of NAD^+^ [8]. We found that the deprivation of lactate by two inhibitors led to an embryo to blastocyst development failure which arrested at the 2-cell stage (Fig. 1D and E), and also found that the potential toxicity of the inhibitors, which may result in this developmental arrest, was excluded by TUNEL staining (Fig. S1E). The FiLa sensor showed the treatment resulted in little or no lactate in the nucleus of 2-cell embryos (Fig. 1F). Considering that the developmental arrest mainly happens at the 2-cell stage, to further determine at which stages lactate is required, we deprived lactate through Gne140 treatment at the late zygote (24 h post-hCG) and 4-cell (60 h post-hCG) stages (Fig. 1G); development failed at the 2-cell stage when lactate was deprived at 24 h post-hCG, but not 60 h (Fig. 1H and I), suggesting the role of lactate in ZGA. Together, these results demonstrate that lactate is essential for the development of mouse embryos beyond the 2-cell stage.

### Lactate deprivation results in failure of major ZGA in early embryos of mice

**Figure 2.**
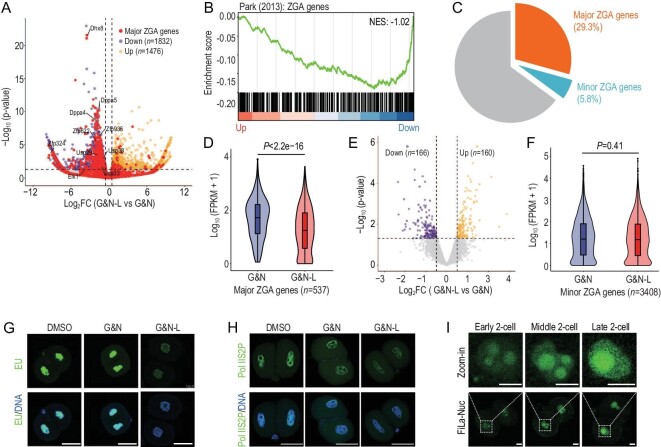
Loss of lactate results in failure of major ZGA in early embryos of mice. (A) RNA-seq analysis of late 2-cell embryos from the G&N and G&N-L groups. Volcano plot showing changes in gene expression. Yellow and purple dots indicate upregulated and downregulated genes, and red dots indicate major ZGA genes. (B) Gene set enrichment analysis (GSEA) of ZGA genes shows preferential downregulation in late 2-cell embryos from the G&N-L group. (C) Fractions of minor ZGA, major ZGA, and other coding genes among the downregulated genes in (A). (D) Violin plot showing the significant downregulation of major ZGA genes in the G&N-L group. (E) RNA-seq analysis of early 2-cell embryos from the G&N and G&N-L groups. Volcano plot showing changes in gene expression. Yellow and purple dots indicate upregulated and downregulated genes. (F) Violin plot showing that the expressions of minor ZGA genes had no significant change between the G&N and G&N-L groups. (G) 5ʹ-Ethynyluridine (EU) staining showing a remarkable decrease in total *de novo* transcripts in late 2-cell embryos of the G&N-L group. Scale bars, 50 μm. (H) IF of Pol II Ser2 of late 2-cell embryos showing a remarkable decrease in the G&N-L group. Scale bars, 50 μm. DMSO, mKSOM culture medium plus DMSO; G&N, mKSOM culture medium plus Gne140 and NMN; G&N-L, mKSOM culture medium without lactate plus Gne140 and NMN. (I) Fluorescence images of mouse 2-cell embryos expressing FiLa in the nucleus. Scale bars, 20 μm. Differences between means were calculated using two-tailed Student's *t*-test.

To investigate the mechanism by which lactate influences early embryo development in mice, we first performed RNA sequencing (RNA-seq) on late 2-cell embryos with deprivation of lactate at 24 h post-hCG. Unsupervised hierarchical clustering (UHC) separated the transcriptomic patterns of the lactate-deprived group from the control group (Fig. S2A), and we identified the differentially expressed genes (DEGs; Fig. 2A and Table S1). KEGG analysis showed that the downregulated genes in the lactate-deprived group were enriched in ZGA-related pathways, such as RNA polymerase, spliceosome, and ribosome (Fig. S2B). ZGA genes were significantly downregulated (NES = −1.02, Fig. 2B) by GSEA. A previous study classified ZGA genes into minor and major ZGA genes based on their expression, mainly at the early or late 2-cell stages [13]. Notably, 29.3% of the downregulated genes (*n* = 537) were major ZGA genes (Fig. 2C), showing significant downregulation through deprivation of lactate (Fig. 2D), including the *Dppa* and *Usp* families (Fig. 2A). To test whether minor ZGA is affected by the deprivation of lactate, we performed RNA-seq on early 2-cell embryos (30 h post-hCG) with lactate deprivation at 24 h post-hCG, and found that the expressions of seldom genes were significantly affected (Fig. 2E; Table S2), while the expression level of minor ZGA genes showed no significant difference (Fig. 2F). To confirm the failure of major ZGA, total *de novo* transcripts were detected via 5ʹ-ethynyluridine (EU) staining; the EU signal was remarkably decreased in the lactate-deprived embryo (Fig. 2G), and also in the enrichment of Pol II Ser2P in the nucleus (Fig. 2H). In addition, the FiLa sensor showed apparently high lactate levels in the nucleus at the late 2-cell stage when major ZGA occurs (Fig. 2I). Together, these results demonstrate that major ZGA is severely impaired in the absence of lactate.

### H3K18lac is reduced by deprivation of lactate and affects major ZGA

**Figure 3.**
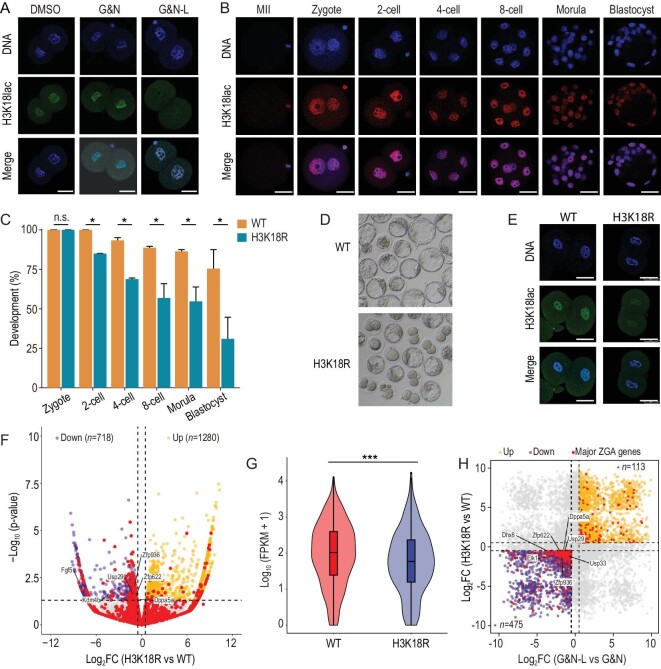
H3K18lac is reduced by deprivation of lactate and affects major ZGA. (A) IF of late 2-cell embryos shows that H3K18lac is remarkably decreased in the G&N-L compared to the other groups. DMSO, mKSOM culture medium plus DMSO; G&N, mKSOM culture medium plus Gne140 and NMN; G&N-L, mKSOM culture medium without lactate plus Gne140 and NMN. Scale bars, 50 μm. (B) IF of H3K18lac during the development of preimplantation embryos developing *in vivo* in mice. Representative images are shown. Scale bars, 50 μm. (C and D) Development rates of embryos with overexpressions of wide type (WT) and mutated H3 mRNAs (H3K18R). Error bars are SEM. n.s. means not significant. *P < 0.05. (E) IF of late 2-cell embryos showing that H3K18lac is decreased by overexpressing H3K18R mRNAs. Scale bars, 50 μm. (F) RNA-seq analysis of late 2-cell embryos with overexpressions of wide type (WT) and mutated H3 mRNAs (H3K18R). Volcano plot shows changes in gene expression. Yellow and purple dots indicate upregulated and downregulated genes, and red dots indicate major ZGA genes. (G) Violin plot showing the significant downregulation of major ZGA genes in the H3K18R mRNAs-overexpressed group. ***P < 0.001. (H) Comparative analysis of the RNA-seq data of the lactate-deprived and H3K18R-mutated embryo. Yellow and purple dots indicate commonly upregulated and downregulated genes, respectively. Red dots represent the major ZGA genes. Differences between means were calculated using two-tailed Student's *t*-test.

Lactate is the substrate for protein lactylation [11]. Considering that the high level of lactate was enriched in the nuclei of 2-cell embryos, we focused on the changes in histone lactylations. By IF analysis of 11 specific histone lactylation sites, we found that the H3K18lac level was markedly reduced in the 2-cell embryos of the lactate-deprived group (Fig. 3A and Fig. S3A). Furthermore, through IF analysis, we observed that H3K18lac was dramatically changed during mouse preimplantation development *in vivo* and *in vitro* (Fig. 3B and Fig. S3B). In contrast to the weak signal in MII oocytes, H3K18lac was established in zygotes and showed a strong H3K18lac signal in the nuclei of 2-cell embryos, especially at the late 2-cell stage (Fig. 3B and Fig. S3B, S3C). To further detect the role of H3K18lac on mouse preimplantation development, we injected mutant H3K18R mRNAs of H3.1, H3.2 and H3.3 into early zygotes at 14 h post-hCG. The loading of H3 on the nuclei in the injected groups was normal as confirmed by IF (Fig. S3D). We found the mixture of the mutant H3K18R mRNAs injection, but not the wildtype mRNAs, resulted in developmental arrest at the 2-cell stage (Fig. 3C and D), suggesting the important role of the lysine 18 sites. As expected, the H3K18lac enrichment was decreased by the overexpression of mutant H3K18R mRNAs mixture (Fig. 3E). In addition to lactylation, the acetylation of the lysine 18 sites is also an important active modification in vertebrates [14]. IF showed that H3K18ac was mainly established at the 8-cell stage and no remarkable signal was observed in 2-cell embryos (Fig. S3E). The results suggest that the developmental arrest by overexpression of mutant H3K18R mRNAs, at least in part, can be contributed to the failure of H3K18lac establishment. RNA-seq data showed the overexpression of mutant H3K18R mRNAs also led to the failed expressions of some major ZGA genes, such as *Dppa5a, Usp29* and *Kdm4b* (Fig. 3F and G; Table S3). In addition, the comparative analysis of the transcriptomes of the lactate-deprived and H3K18R-mutated embryos exhibited 475 commonly downregulated major ZGA genes (Fig. 3H). Therefore, we suggest the role of lactate-derived H3K18lac in regulating major ZGA.

### Dynamics of H3K18lac during the development of preimplantation embryos in mice

**Figure 4.**
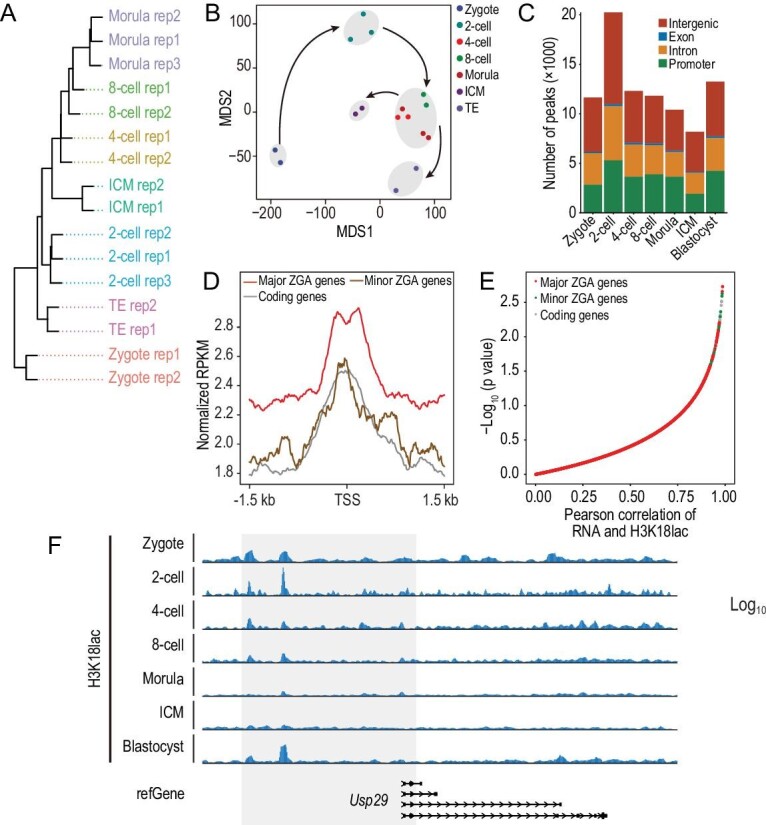
H3K18lac genome-wide distribution in preimplantation embryos of mice. (A) Unsupervised clustering of H3K18lac signals among the embryos at the stages indicated. (B) MDS analysis of H3K18lac profiles. Different colors indicate different developmental stages and lineages. ICM, inner cell mass; TE, trophoblast. (C) Fraction of the mouse genome covered by H3K18lac reads at different developmental stages, and percentages of H3K18lac peaks assigned to the promoter, intron, exon, and intergenic regions. (D) Metaplot of H3K18lac enrichment at the promoters of minor and major ZGA, and other coding genes at the late 2-cell stage. (E) Pearson correlation analysis of gene expression and H3K18lac enrichment at the gene promoters in mouse preimplantation embryos. Red, green and grey dots represent major ZGA, minor ZGA, and coding genes, respectively. (F) Genome browser view of H3K18lac signals at the *Usp29* locus during the development of the preimplantation embryo.

To figure out how H3K18lac functions on major ZGA, we first used cleavage under targets and tagmentation (CUT&Tag) to generate genome-wide H3K18lac histone modification maps for zygote, late 2-cell, 4-cell, 8-cell, morula, ICM, and TE of blastocyst. The strong correlations between the biological replicates confirmed data quality (Fig. S4A). Hierarchical clustering distinguished the stages (Fig. 4A), and multiple dimensional scaling (MDS) separated the late 2-cell embryo from the embryos at other stages, showing the specific manner of H3K18lac at the late 2-cell stage (Fig. 4B). Dramatic changes of H3K18lac distributions were observed (Fig. S4B), and we found that H3K18lac correlated with H3K27ac, and excluded to H3K27me3 (Fig. S4C). The genomic coverage analysis revealed that the H3K18lac modification was present at the highest level in the 2-cell embryos, and more peaks were enriched at the promoter at late 2-cell stage than those at other stages (Fig. 4C). More importantly, the peaks of H3K18lac at the promoter regions of major ZGA genes were significantly higher than those of minor ZGA genes and other coding genes (Fig. 4D). In addition, the expressions of most major ZGA genes exhibited high correlation with H3K18lac (Fig. 4E), such as *Usp29* (Fig. 4F). We also found the downregulated genes in the lactate-deprived group showed significantly high H3K18lac enrichment comparing to the upregulated genes (Fig. S4D), suggesting that alteration in transcriptome of the lactate-deprived 2-cell embryos may be associated with the loss of H3K18lac. Taken together, these data clearly show that H3K18lac is enriched in the promoters of major ZGA genes in 2-cell embryos, and may correlate with their expressions.

### H3K18lac promotes the expression of major ZGA genes

**Figure 5.**
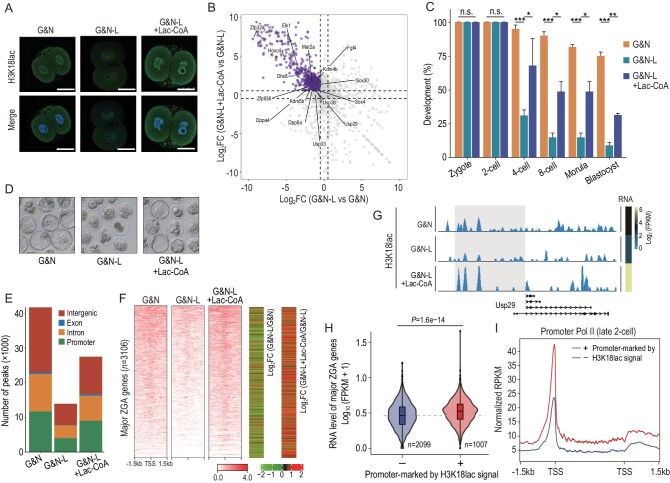
Lac-CoA can rescue the abnormal phenotypes caused by deprivation of lactate. (A) IF of H3K18lac in late 2-cell embryos from the groups indicated. Scale bars, 50 μm. (B) RNA-seq analysis showing the expressions of major ZGA genes rescued by the addition of 1 mM Lac-CoA. Purple dots indicate the rescued major ZGA genes. (C and D) Development rates of embryos from the groups indicated. Three experimental replicates were performed. Error bars are SEM. n.s. means not significant. *P < 0.05; **P < 0.01; ***P < 0.001. (E) Fraction of the mouse genome covered by H3K18lac reads in the groups indicated, and percentages of H3K18lac peaks assigned to the promoter, intron, exon, and intergenic regions. (F) Heatmap (left panel) of H3K18lac signals ranked by their relative changes among the groups indicated. Heatmap (right panel) showing the expression changes of major ZGA genes between the groups indicated. (G) Genome browser view of H3K18lac signals at the *Usp29* locus of late 2-cell embryos from the groups indicated. G&N, mKSOM culture medium plus Gne140 and NMN; G&N-L, mKSOM culture medium without lactate plus Gne140 and NMN; G&N-L + Lac-CoA, zygotes injected into 1 mM Lac-CoA and cultured in mKSOM medium without lactate plus Gne140, NMN. (H) Violin plot showing the significantly upregulated expressions of major ZGA genes with H3K18lac enrichment. (I) Metaplot showing the higher Pol II loading on the locus of major ZGA genes with H3K18lac enrichment. Differences between means were calculated using two-tailed Student's *t*-test.

Lac-CoA has been shown to be the substrate for histone lactylation [11]. We found injection of 1 mM Lac-CoA into zygotes deprived of lactate at 24 h post-hCG could rescue the reduced H3K18lac level (Fig. 5A), and the failed expressions of major ZGA genes checked by RNA-seq, including *Dppa* and *Usp* genes (Fig. 5B; Table S4), and the developmental arrest (Fig. 5C and D). To investigate the role of H3K18lac on major ZGA, we generated genome-wide H3K18lac maps of late 2-cell embryos from the group with Lac-CoA addition using CUT&Tag. Compared to the lactate-deprived embryos, the average enrichment of H3K18lac signals was remarkably recovered by Lac-CoA, also at the promoter regions (Fig. 5E). Importantly, Lac-CoA could rescue the H3K18lac enrichment in promoter regions of the majority of major ZGA genes (Fig. 5F). The RNA-seq and CUT&Tag data showed that the H3K18lac enrichment in promoter regions was positively correlated with the expression levels of major ZGA genes (Fig. 5F), such as *Usp29* (Fig. 5G). Furthermore, the major ZGA genes with high H3K18lac enrichment (1 004 vs 2 099) showed significantly higher expressions (Fig. 5H), and Pol II loading on the TSS and TES regions (Fig. 5I), suggesting H3K18lac may facilitate Pol II loading to promote expressions of major ZGA genes. Collectively, our results indicate that lactate regulates major ZGA, alternatively via H3K18lac.

### H3K18lac is temporally concomitant with major ZGA in early embryos of humans

**Figure 6.**
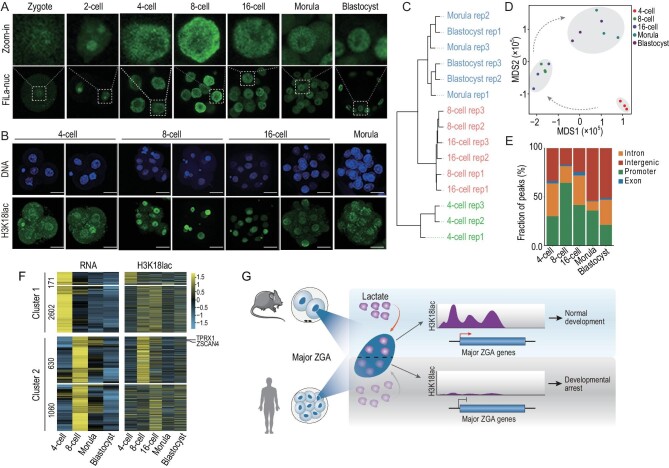
H3K18lac-enriched ZGA markers in human 8-cell embryos. (A) Fluorescence images of human embryos from different stages expressing FiLa in nuclei. (B) IF of H3K18lac of human 4-cell, 8-cell, 16-cell, and morula embryos. Scale bars, 50 μm. (C) Unsupervised clustering of H3K18lac signals among the embryos at the stages indicated. (D) MDS analysis of samples in the indicated stages. Different colors indicate different developmental stages. (E) Ratio of fraction of the human genome covered by H3K18lac reads in the indicated developmental stages, and percentages of H3K18lac peaks assigned to the promoter, intron, exon, and intergenic regions. (F) Heatmaps of H3K18lac enrichment (right panel) in 8-cell signature genes indicated in human embryos; left panel, corresponding RNA levels. (G) Lactate in nuclei when major ZGA occurs in humans and mice during preimplantation development and a model of lactate-derived H3K18lac regulating major ZGA.

The finding that lactate via its derived H3K18lac regulates major ZGA, and for further embryonic development of mouse embryos, led us to investigate whether a conserved mechanism might operate in human embryos. To this end, we were granted access to a small number of human embryos from fertilization clinics through a fully consented and documented process. As in mice, we found the high level of lactate enrichment in nuclei at the 8-cell stage (Fig. 6A and Fig. S5A, S5B), when major ZGA bursts occurred in humans. IF showed that the high H3K18lac signals were observed in the 8-cell embryo, while the signals were low in the 4-cell embryos and morula (Fig. 6B). To investigate the role of H3K18lac in human ZGA, we performed genome-wide H3K18lac profiling of human embryos. Because of the small number of samples, we developed a single-cell CUT&Tag (scCut&Tag) approach. First, the approach was performed on H3K4me3 using single 2-cell embryos of mice. The H3K4me3 signals detected by scCut&Tag showed high correlation and similar pattern with those detected by ULI-NChIP and Cut&Tag using bulk cells in our previous study [15] and the study shown in Fig. S6A, S6B. We then titrated a range from 100 to 1 2-cell mouse embryos to validate the approach. We found strong correlations between the number of embryos and the H3K18lac signals even at the single 2-cell embryo level (Fig. S6C), and the chromatin landscapes of the ultra-low samples closely matched profiles generated in bulk samples, indicating the accuracy of the approach (Fig. S6D). Then, we performed detection on human embryos from the 4-cell to blastocyst stages. Hierarchical clustering analysis could distinguish samples at different stages (Fig. 6C), and MDS separated the 8-cell and 16-cell embryos from the embryos at other stages (Fig. 6D), showing the specific role of H3K18lac on ZGA in humans. Genomic coverage analysis revealed the highest ratio of H3K18lac peaks enrichment at the promoter regions in 8-cell embryos (Fig. 6E). Furthermore, we classified the 8-cell embryo signature genes classified by a recent study [16] into those highly expressed in 4-cell (cluster I) and 8-cell (cluster II) embryos, respectively. Importantly, the promoter regions of hundreds of these genes, including human ZGA markers (such as *ZSCAN4* and *TPRX1*), were highly enriched with H3K18lac peaks that correlated with their expressions (Fig. 6F). The results reveal the expressions of the key human 8-cell embryo signature genes are associated with H3K18lac enrichment.

## DISCUSSION

Cell metabolism provides not only energy but also substrates or cofactors for epigenetic remodeling, which is important in preimplantation embryo development in mammals. Some metabolic changes are attributed to changes in chromatin status and therefore are associated with developmental progression [4,17]. In this study, we observe lactate is highly enriched in the nuclei of early embryos when major ZGA occurs in humans and mice, and, in absence of lactate, mouse embryos fail in major ZGA with developmental arrest at the 2-cell stage, and show the role of lactate-derived H3K18lac in major ZGA (Fig. 6G).

The preimplantation embryo takes up nutrients from the oviductal fluid. The developmental program is recapitulated for *in vitro* culture of embryos in a defined medium, which contains most of the components of oviductal fluid, including pyruvate, lactate, and glucose [18,19]. Pyruvate is indispensable for the development of mouse embryos beyond the 2-cell stage [20], and glucose is essential for the morula-to-blastocyst transition [21]. Lactate supports the development of early embryos of mice after the 2-cell stage, but it is readily oxidized by 1- and 2-cell embryos [10]. However, because the LDH-mediated conversion of pyruvate into lactate is at a high level in the cleavage stage, removing lactate from the culture medium had no significant effect on *in vitro* development of preimplantation mouse embryos. KSOM contains lactate at 20 mM, indicating that a large amount of lactate is consumed at the early stages. In the zygote stage, pyruvate uptake and oxidation are significantly reduced in the presence of a high level of lactate, suggesting that lactate is converted into pyruvate [10]. Therefore, the interconversion of lactate and pyruvate may promote embryo adaptation to the microenvironment, particularly in the cleavage stages. By inhibiting the production and uptake of lactate, we found that preimplantation development of the mouse embryo is blocked in the 2-cell stage. Therefore, lactate is a key player in early developmental stages. Indeed, both lactate and pyruvate are indispensable for embryo development beyond the 2-cell stage, and they may regulate preimplantation embryo development by different mechanisms.

It has been shown that a number of enzymatically active mitochondrial enzymes associated with the TCA cycle are transiently and partially localized to the nucleus during ZGA. Pyruvate is essential for this nuclear localization [20]. In the study, we also show that LDH in the glycolysis pathway is localized in the nucleus, revealing the important role of the lactate mechanism in the nucleus in ZGA. The nuclear lactate level in the inhibitor treatment groups were already dramatically lower than the control group, indicating that a certain level of lactate in the nucleus of 2-cell embryos is indispensable for normal preimplantation development in mice. Thus, the nuclear lactate level is decreased by the inhibitor treatment, but the lactate uptake from culture medium is sufficient to support preimplantation development. Lactate can feed the TCA cycle in the mitochondria and also lactylate histones in the nucleus [11]. The interesting finding in the study is that lactate is temporarily localized to the nuclei in human and mouse embryos when major ZGA occurs, indicating the lactate-mediated epigenetic remodeling functions during ZGA. Histone lactylation directly promotes gene transcription [11]. In the late phase of M1 (proinflammatory) macrophage polarization, increased histone lactylation in promoter regions induces the expression of homeostatic genes and facilitates acquisition of the M2 (anti-inflammatory)-like phenotype [11]. To date, 28 lactylation sites on core histones have been identified, including H3K4, H3K18, H4K12 and H4K5 [22–26]. H3K18lac promotes the expression of YTHDF2, which recognizes and enhances the degradation of m6A-modified PER1 and TP53 mRNAs, thus driving oncogenesis [27]. In the study, we find that deprivation of lactate leads to a remarkable loss of H3K18lac in mouse 2-cell embryos. And the developmental arrest and failure of the major ZGA induced by lactate depletion, were recapitulated by the overexpression of mutant H3K18R mRNAs, and more importantly, recovered by addition of Lac-CoA, indicating the important role of H3K18lac in major ZGA. Investigation of the dynamic regulation of H3K18lac during the preimplantation development of mammalian embryos facilitates functional evaluation of lactate signaling pathways. For the first time, we profiled H3K18lac in the preimplantation embryos of humans and mice. This modification is enriched in major ZGA genes, and contributed to their expressions. Lactate can also activate the expression of ZGA markers, such as Zscan4 and MERVL, in mouse ESCs to induce the 2-cell-like cells. Thus, we also propose a critical function of lactate-mediated epigenetic remodeling in regulating totipotency.

In summary, lactate is indispensable for the preimplantation development of mammalian embryos, and we show the role of lactate-derived H3K18lac in major ZGA. This study was a preliminary exploration of lactate metabolism-epigenetic relationships in mammalian embryos. It would be interesting to determine whether the mechanism of normal development presented here has clinical relevance for infertility, which is often associated with metabolic disorders.

## MATERIALS AND METHODS

Detailed materials and methods are available in the Supplementary Data. This study was approved by the Institutional Review Board (IRB) of Chongqing Health Center for Women and Children (2019-603), China, in accordance with the measures of the People's Republic of China on the Administration of Human Assisted Reproductive Technology and the Helsinki Declaration. The research followed the principles of the Human Embryonic Stem Cell Ethics issued by the MOST and MOH and was regularly reviewed by the Medical Ethics Committee of Chongqing Health Center for Women and Children.

## Supplementary Material

nwad295_Supplemental_Files

## Data Availability

Publicly available datasets analyzed in this work are available in GEO, the GSE ID shown below: Pol II (GSE135457), H3K4me3/H3K27ac (GSE72784, GSE185653), H3K4me3/H3K27me3 (GSE73952), H3K9me3 (GSE98149). All sequencing data of mouse embryos generated in this study have been deposited in GEO under accession GSE234027. All sequencing data of human embryos generated in this study have been deposited in GSA-Human under the accession number HRA004614 in PRJCA017054.
