# Supplementary material for: Lactate regulates major zygotic genome activation by H3K18 lactylation in mammals
Source: Natl Sci Rev. 2023 Nov 20;11(2):nwad295. doi: 10.1093/nsr/nwad295 (PMC10849771; doi:10.1093/nsr/nwad295)
Supplement: nwad295_Supplemental_Files [file nwad295_supplemental_files.zip › Supplementary_figures.docx]

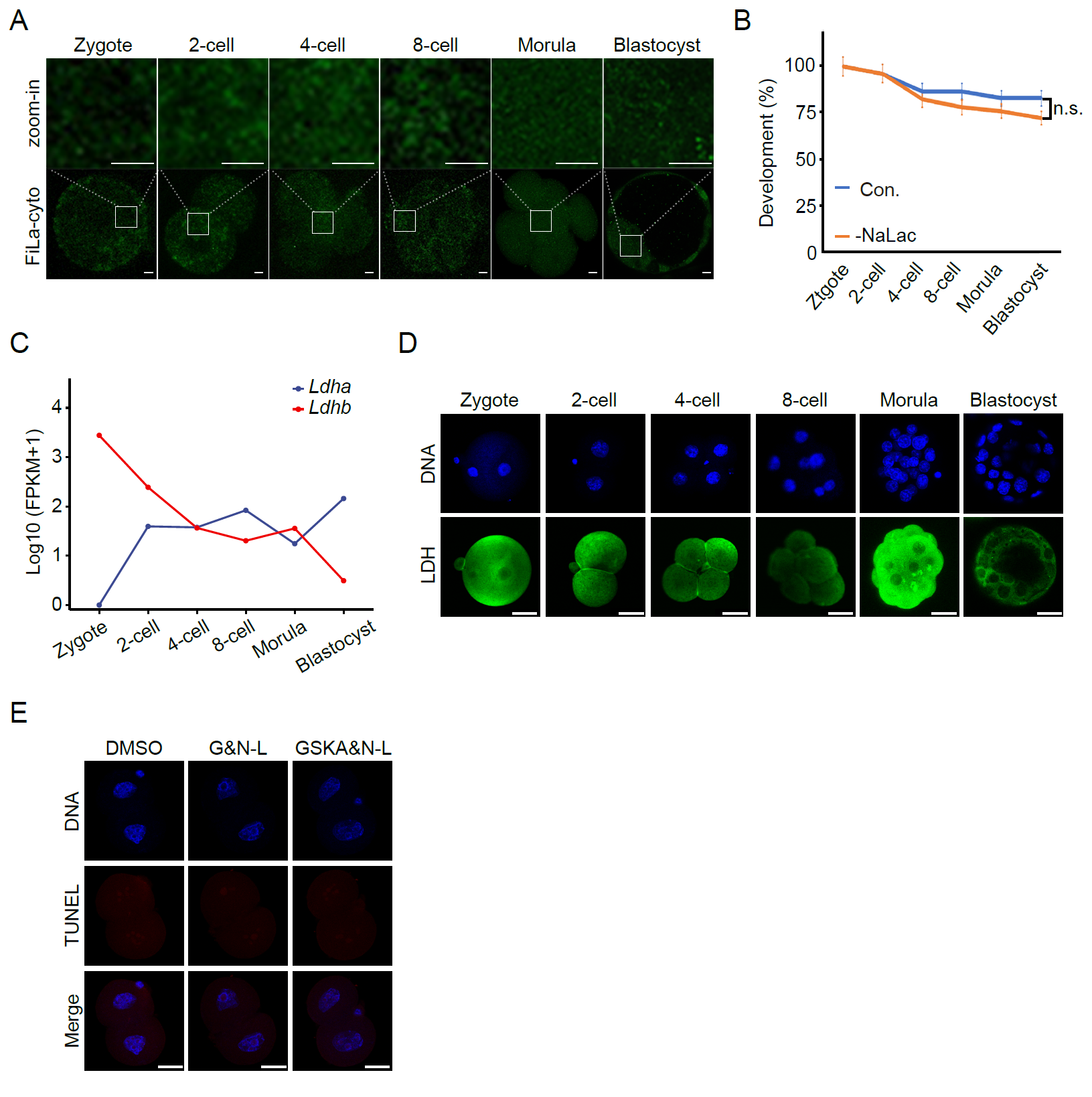


**Supplementary Fig. 1. Lactate is important for mouse early embryo. (A)** Fluorescence images of mouse embryos from different stages expressing FiLa in the cytoplasm. **(B)** Developmental rates of embryos in control and -Nalac groups. n.s. means no significant. **(C)** Expression patterns of *Ldha* and *Ldhb* according to RNA-seq data from mouse preimplantation embryos. **(D)** IF of LDH at various stages during preimplantation embryo development in mice. Scale bars, 50 μm. **(E)** TUNEL staining of mouse embryos at 2-cell stage from different groups. Scale bars, 50 μm.


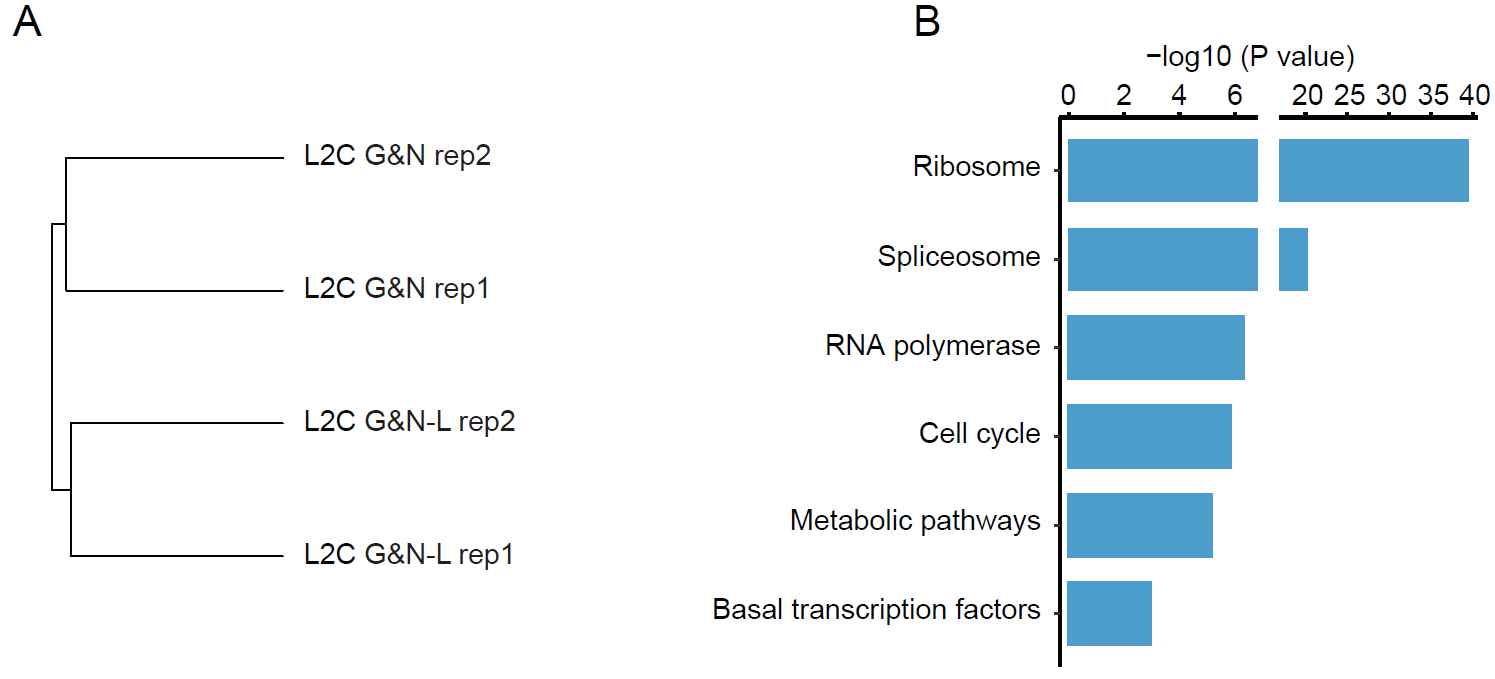


**Supplementary Fig. 2. Lactate is necessary for major ZGA in mouse early embryo. (A**) Unsupervised clustering of gene expression between groups indicated. **(B**) KEGG analysis of the downregulated genes in G&N-L group. G&N, mKSOM culture medium plus Gne140 and NMN; G&N-L, mKSOM culture medium without lactate plus Gne140 and NMN.


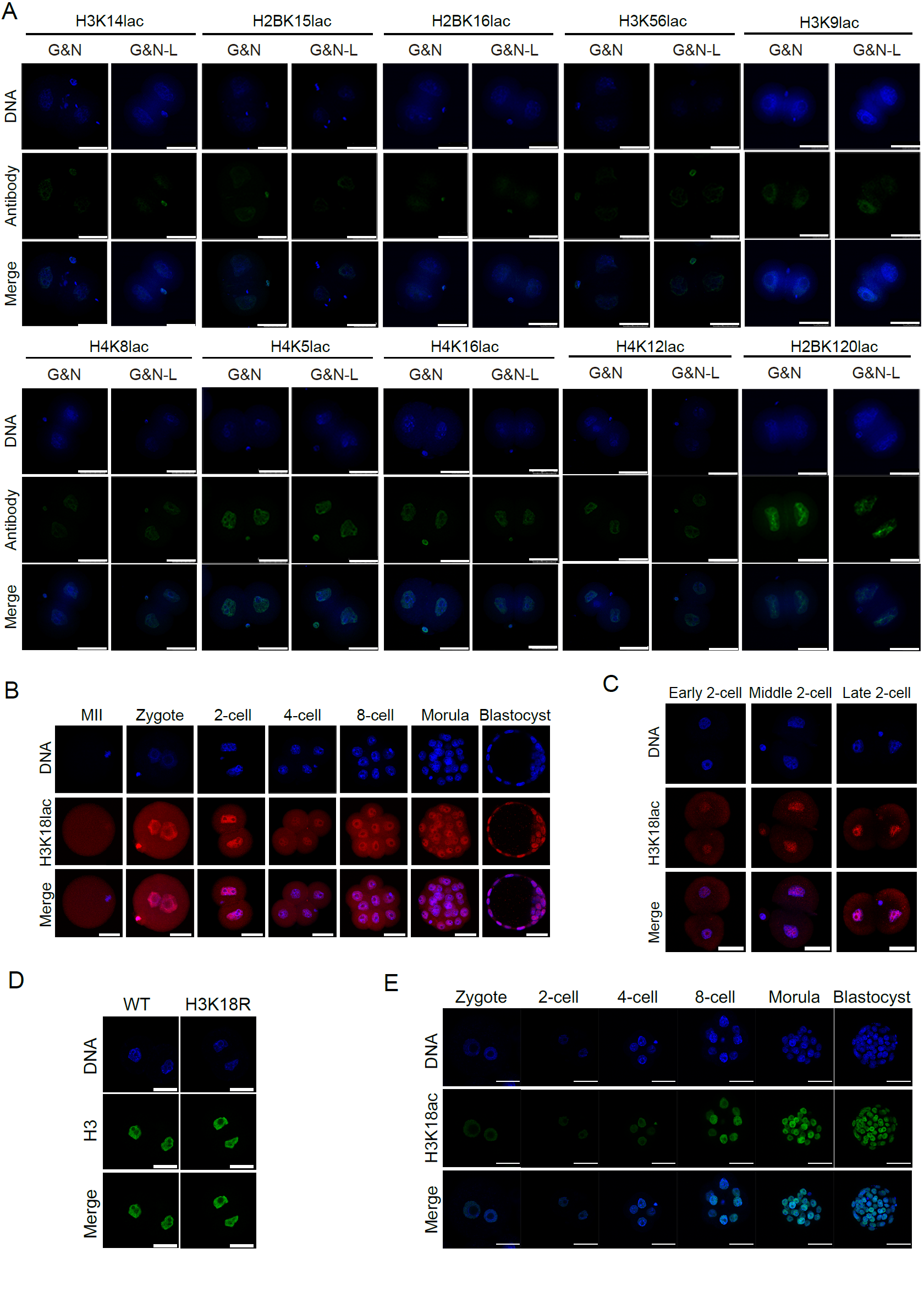


**Supplementary Fig. 3. Histone lactylations affected by deprivation of lactate. (A**) IF of specific histone lactylation sites with and without lactate. **(B**) IF of H3K18lac during the development of preimplantation embryos developing *in vitro* in mice. Representative images are shown. **(C)** IF of H3K18lac at early-, middle-, and late-2-cell embryos in mice. **(D)** IF of late 2-cell embryos showing successful overexpression of wildtype and mutant H3. **(E**) IF of H3K18ac during mouse pre-implantation embryo development. Scale bars, 50 μm.


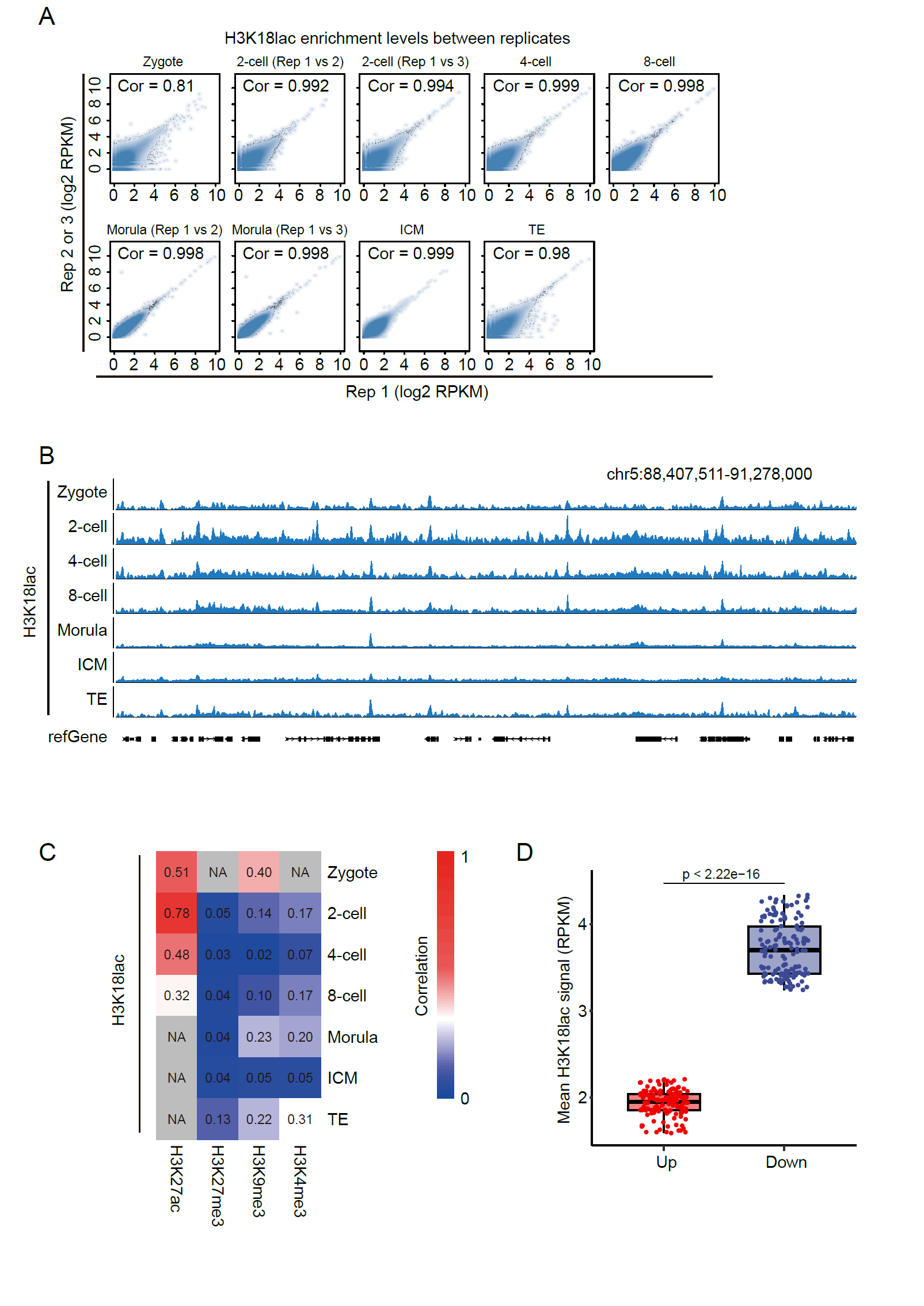


**Supplementary Fig. 4 Dynamics of H3K18lac during the development of preimplantation embryos in mice. (A**) Strong correlations between the biological replicates confirming the data quality of CUT&Tag. **(B**) Genome-wide landscapes of H3K18lac modification during mouse preimplantation embryo development. **(C**) Cross talks between H3K18lac with H3K4me3, H3K27ac, H3K9me3 and H3K27me3. **(D)** The enrichment of H3K18lac on the promotors of up- and down-regulated genes by the deprivation of lactate. Differences between means were calculated using two-tailed Student’s t-test.


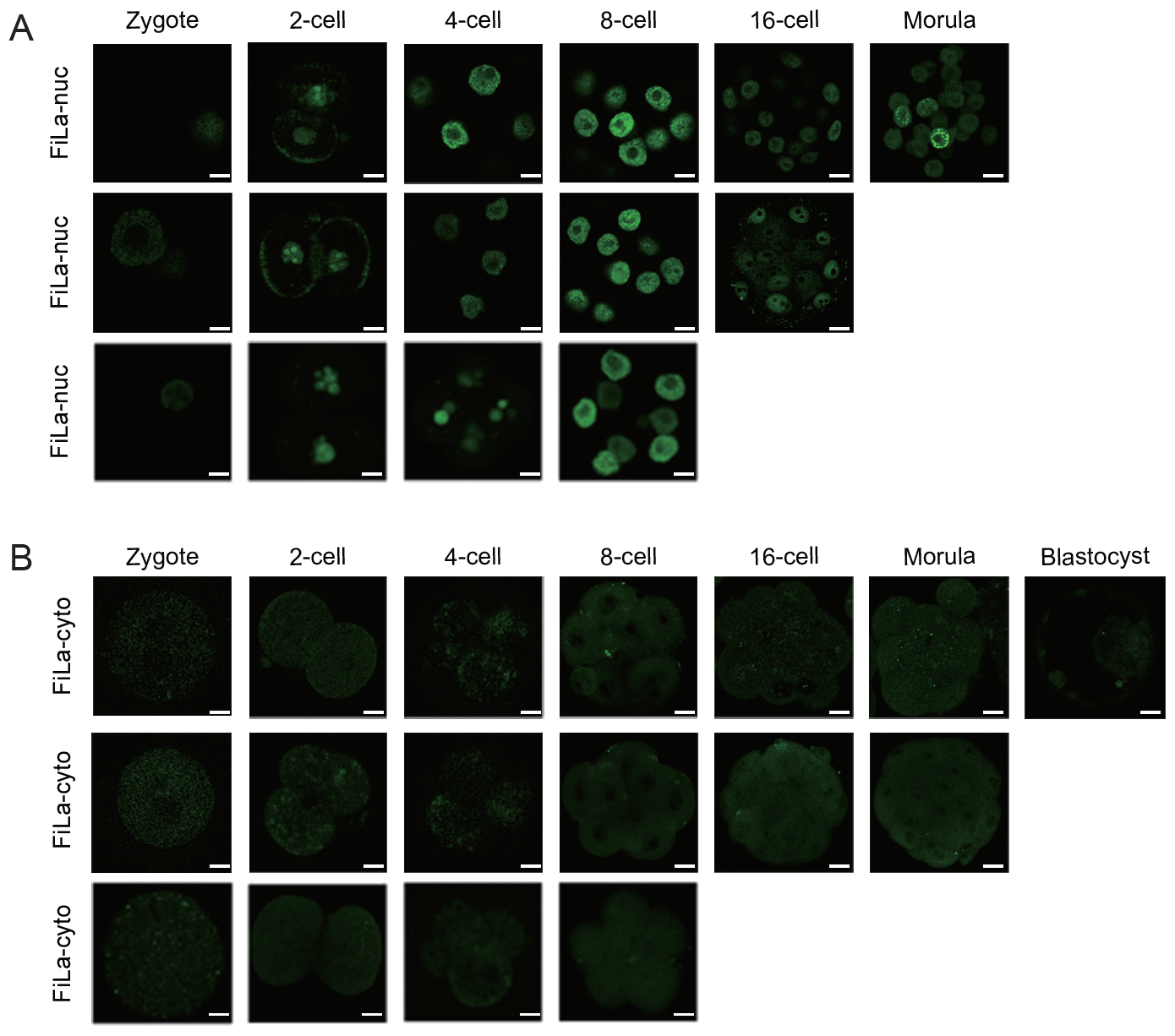


**Supplementary Fig. 5.** **Fluorescence images of human embryos from different stages expressing FiLa in nuclei (A) and cytosol (B).** Scale bars, 20 μm.


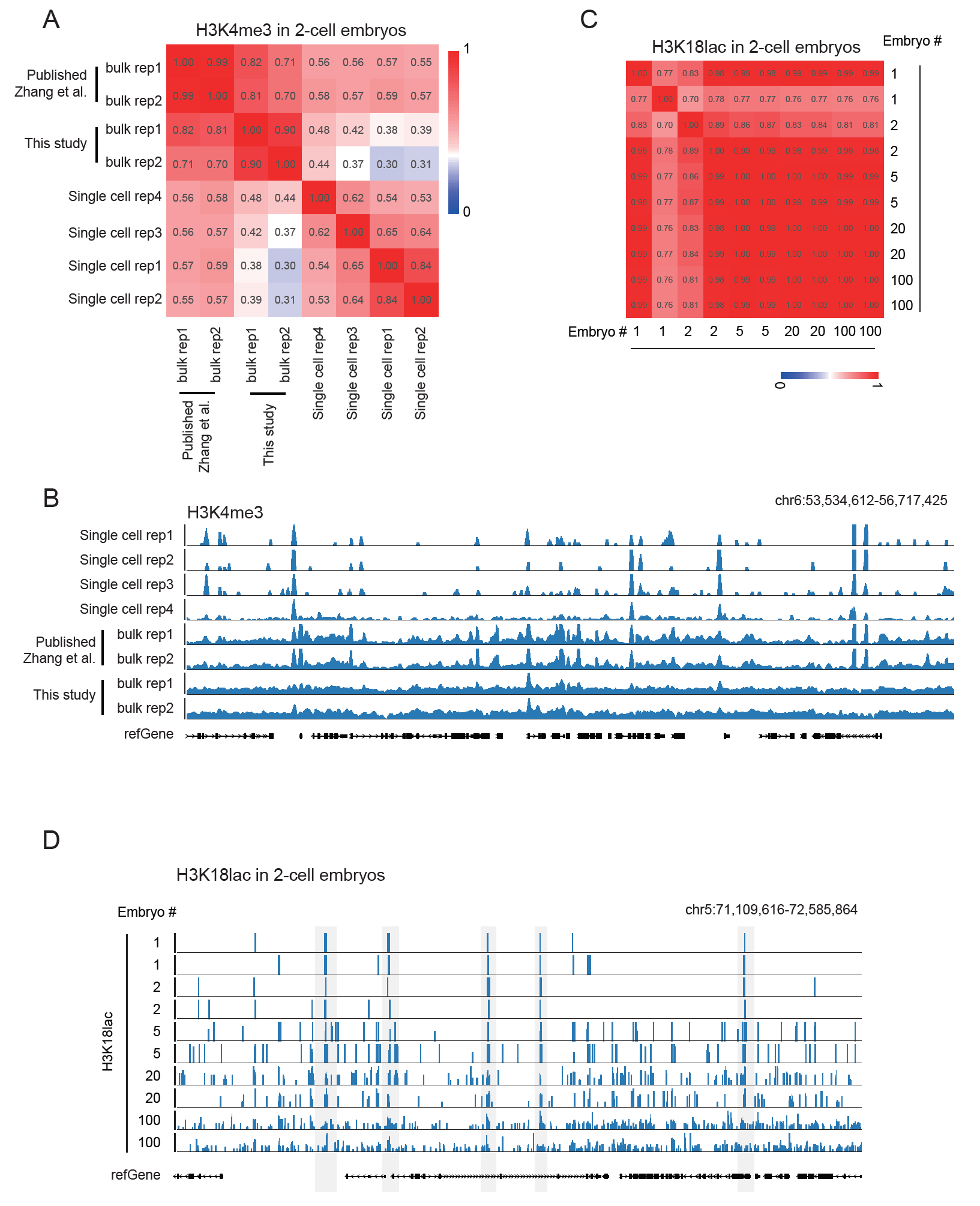


**Supplementary Fig. 6. Validation of the scCUT&Tag approach. (A)** Strong correlations between scCUT&Tag and ChIP-seq of H3K4me3. **(B)** Genome-wide landscapes of H3K4me3 histone modification detected by scCUT&Tag. **(C)** Strong correlations between the number of embryos and the H3K18lac signals at the single 2-cell embryo level. **(D)** Ultra-low sample chromatin profiles of H3K18lac signals matched those of bulk samples.
