# Supplementary material for: Lactate regulates major zygotic genome activation by H3K18 lactylation in mammals
Source: Natl Sci Rev. 2023 Nov 20;11(2):nwad295. doi: 10.1093/nsr/nwad295 (PMC10849771; doi:10.1093/nsr/nwad295)
Supplement: nwad295_Supplemental_Files [file nwad295_supplemental_files.zip › Supplementary Materials and methods.docx]

**Supplementary Materials and methods**

**Ethics statement.**

This study was approved by the Institutional Review Board (IRB) of Chongqing Health Center for Women and Children (2019-603), China, in accordance with the measures of the People’s Republic of China on the Administration of Human Assisted Reproductive Technology and the Helsinki Declaration. The research followed the principles of the Human Embryonic Stem Cell Ethics issued by the MOST and MOH and was regularly reviewed by the Medical Ethics Committee of Chongqing Health Center for Women and Children.

**Human early embryo collection.**

Frozen embryos were thawed using a Glass Solution Freeze Set according to the manufacturer’s instructions (Kitazato Corporation). Embryos before the 8-cell stage were cultured in G-1 (Vitrolife) human embryo medium at 37℃ in 6% CO_2_ and 5% O_2_. G-2 (Vitrolife) medium was used from the 8-cell to blastocyst stages. The numbers of embryos used were as follows: two 4-cell, two 8-cell, two 16-cell embryos, and one morula were used for IF; three each of 4-cell, 8-cell, 16-cell morula and blastocyst embryos subjected to scCUT&Tag and 7 zygotes cultured to blastocyst stage were used for FiLa analysis.

**Mice.**

Mice were maintained and handled according to policies approved by Chongqing Health Center for Women and Children. Male and female ICR mice (6–8 weeks old) were purchased from Charles River (Beijing, China).

**Embryo collection.**

Embryos were collected from superovulated female ICR mice treated with 6.5 IU pregnant mare serum gonadotropin (PMSG, ProSpec, Cat HOR-272); after 47–48 h, they were treated with 5 IU human chorionic gonadotropin (hCG, ProSpec, Cat HOR-250) and crossed with male ICR mice. For the *in vitro* embryos, time points of post-hCG injection: 24 h for the late 1-cell stage, 30 h for the early 2-cell stage, 40 h for the middle 2-cell stage, 54 h for the late 2-cell stage, 60 h for the 4-cell stage, 70 h for the 8-cell stage, 78 h for the morula stage, and 94 h for the blastocyst stage. The *in vivo* embryos were collected through flushing the fallopian tubes and uterine horns with M2 medium, time points are as follows: 22h for the zygote stage, 47h for the 2-cell stage, 54h for the 4-cell stage, 66h for the 8-cell stage, 76 h for the morula stage, and 88 h for the blastocyst stage. Embryos in the control group were cultured in modified KSOM medium (95 mM NaCl, 2.5 mM KCl, 0.35 mM KH_2_PO_4_, 0.20 mM MgSO_4_, 25 mM NaHCO_3_, 1.71 mM CaCl_2_, 0.01 mM EDTA, 0.20 mM glucose, 10 mM lactate, and 0.20 mM pyruvate) devoid of amino acids and BSA[28]. Medium osmolarity was maintained using 0.1% polyvinyl alcohol under mineral oil at 37°C in a 5% CO_2_ incubator.

**Treatment of embryos.**

We prepared solutions of GNE-140 (MedChemExpress, Cat HY-100742A) or GSK2837808A (MedChemExpress, Cat HY-100681) in dimethyl sulfoxide (DMSO). NMN (Sigma, Cat N3501) solutions were prepared in water. Solutions were diluted to the desired final concentration in medium (GNE-140, 5 µM; GSK2837808A, 1µM; NMN, 50 µM). Embryos were cultured *in vitro* in modified KSOM medium containing the indicated doses of inhibitors.

**Measurement of lactate level.**

The lactate level was measured using the Amplite™ Colorimetric L-Lactate Assay Kit (AAT Bioquest, Cat AAT-13815) according to the manufacturer’s instructions. Briefly, 5 µL culture medium was collected at 1 h intervals, and the difference between two adjacent times was considered lactate uptake. To measure the lactate level of embryos, 200 embryos were collected from each stage in 3 repeats. To avoid lactate from KSOM, embryos were washed 5 times in PBS. Following the addition of an equal volume of reagent buffer (AAT Bioquest), luminescence was measured to assess the lactate level.

***In vitro* mRNA transcription.**

To prepare mRNAs for microinjection, pUC-GW-Amp-H3.1_K18R, pUC-GW-Amp-H3.2_K18R, pUC-GW-Amp-H3.3_K18R, pUC-GW-Amp-H3.1WT, pUC-GW-Amp-H3.2WT and pUC-GW-Amp-H3.3WT vectors were linearized and purified with phenol-chloroform, followed by ethanol precipitation. Linearized DNAs were transcribed *in vitro* using the mMESSAGE mMACHINE Kit (Invitrogen, Cat AM1344), recovered by lithium chloride precipitation, and resuspended in nuclease-free water. mRNA was frozen and stored at –80°C.

**Microinjection.**

For FiLa analysis, pAAV-CMV-MCS-FiLa, pAAV-CMV-MCS-FiLa-C, pLVX-Nuc-FiLa and pLVX-Nuc-FiLa-C were linearized and *in vitro* transcribed, and 200 ng/µL mRNA was microinjected into human and mouse early zygotes. For overexpression, *H3.1K18R*, *H3.2K18R*, *H3.3K18R*, *H3.1WT*, *H3.2WT* and *H3.3WT* mRNA (200 ng/µL) was microinjected into zygotes. Microinjection was performed using a FemtoJet 4i Microinjector (Eppendorf, Hamburg, Germany) and ELIPSE Ti Micromanipulators (Nikon, Tokyo, Japan). For injection, a glass capillary Femtotip (Eppendorf) was loaded with the mixtures using a Microloader (Eppendorf) and injected into cytoplasm in a drop of M2 medium (Merck). The injection volume was approximately 2-5 pL. The injection pressure was 250 hPa, compensation pressure was 60 hPa, and injection time was 0.7 s. Immediately after microinjection, embryos were cultured at 37°C in 5% CO_2_.

**Immunofluorescence.**

After removing the zona pellucida using acidic operating fluid, mouse embryos were fixed in 4% PFA for 40 min at room temperature, followed by permeabilization in 1% Triton X-100 (93443, 100 mL; Sigma) for 20 min at room temperature. Embryos were blocked in blocking solution consisting of 1% bovine serum albumin (BSA) in phosphate-buffered saline (PBS) for 1 h at room temperature after three washes in washing solution (0.1% Tween-20, 0.01% Triton X-100 in PBS). Embryos were incubated with the indicated antibodies against H3K27ac (Active Motif, Cat 39034), H3K18lac (PTM BIO, Cat PTM-1406RM), LDH (Abcam, ab52488), Pol II Ser 2 (Abcam, ab193468), H3K18ac (Abcam, ab40888), H4K5lac (PTM BIO, Cat PTM-1407RM), H4K8lac (PTM BIO, Cat PTM-1415RM), H2BK15lac (PTM BIO, Cat PTM-1426RM), H3K14lac (PTM BIO, Cat PTM-1414RM), H2BK16lac (PTM BIO, Cat PTM-1424RM), H2BK120lac (PTM BIO, Cat PTM-1423), H4K12lac (PTM BIO, Cat PTM-1411RM), H3K9lac (PTM BIO, Cat PTM-1419RM), H4K16lac (PTM BIO, Cat PTM-1417RM), H3K56lac (PTM BIO, Cat PTM-1421RM) overnight at 4°C. The next day, the embryos were washed in washing solution and incubated with the secondary antibodies (A10040; Invitrogen) for 1 h at room temperature. After staining with Hoechst, the embryos were washed in washing solution and imaged using an inverted confocal microscope (TCS SP8; Leica, Wetzlar, Germany); image analysis was performed using LAS X software (Leica).

**EU staining.**

Incorporated EU was detected using the click-iT RNA Alexa Fluor 488 Imaging Kit (Invitrogen, Cat C10329). EU (10 M, final concentration) was added to cultured mouse embryos at 40 h post-hCG. After removal of the zona pellucida using acidic Tyrode’s solution, 2-cell embryos of mice were washed twice for 5 min each in washing solution. Embryos were fixed with 4% PFA in 1× PBS for 30 min, followed by permeabilization with normal permeabilizing solution. After incubated with Click-iT reaction cocktail, the embryos were washed in Click‑iT reaction rinse buffer. Embryos were imaged using an inverted confocal microscope (TCS SP8; Leica, Wetzlar, Germany); image analysis was performed in LAS X software (Leica).

**Embryo toxicity detection.**

To assess the potential toxicity of inhibitors on embryos, cell apoptosis was tested utilizing terminal deoxynucleotidyl transferase–mediated deoxyuridine triphosphate (dUTP) nick end labeling (TUNEL) assay kit (C1090, Beyotime) according to the manufacturer’s instructions. Specifically, mouse embryos were fixed in 4% PFA for 30 min at room temperature, followed by permeabilization in 0.5% Triton X-100 (93443, 100 mL; Sigma) for 15 min at room temperature. Embryos were washed three times for 5 min each in washing solution, and were incubated with the TUNEL solution. Embryo apoptosis was then examined by immunofluorescence staining.

**RNA-seq.**

RNA-seq libraries were prepared as described previously. Briefly, 5 embryos were used per group, and three replicates were performed for each group. Embryos were washed three times in 0.5% BSA-PBS solution. cDNA was amplified using the Phusion Hot Start II High-Fidelity PCR Master Mix (Thermo Fisher). Library preparation was performed using the NEBNext Ultra II DNA Library Prep Kit (New England Biolabs) according to the manufacturer’s instructions. Libraries were sequenced using the NovaSeq 6000 (Illumina, San Diego, CA) according to the manufacturer’s instructions.

**RNA-seq data quality control, processing, and analysis.**

For RNA-seq analysis of embryos, FastQC was performed for Illumina reads. We used Trim Galore software to discard low-quality reads, trim adaptor sequences, and eliminate poor-quality bases. Next, we downloaded the mouse reference genome (genome assembly: mm39) from the UCSC database and used HISAT2 software for read alignment. The gene-level quantification approach was used to aggregate raw counts of mapped reads using the featureCounts tool. Gene expression was quantified by calculating the normalized fragments per kilobase of transcript per million mapped reads (FPKM) value. Next, we used the R package DESeq2 for differential gene expression analysis. KEGG analysis of DEGs was performed using the KOBAS online tool (<http://kobas.cbi.pku.edu.cn/kobas3/>). For GSEA, DEGs were identified as above. Significantly DEGs were ranked and subjected to pre‑ranked GSEA analysis.

**CUT&Tag.**

CUT&Tag was performed using the Hyperactive In-Situ ChIP Library Prep Kit for Illumina (Vazyme Biotech). Embryos were incubated in 50 µL antibody buffer; 0.5 μg antibody was added and the mixture was incubated at 4℃ overnight. After washing twice with dig-wash buffer, 50 µL dig-wash buffer with 0.2 µg secondary antibody was added and the mixture was incubated at room temperature for 1 h. After washing twice with 100 µL dig-wash buffer, 1 µL pG-Tn5 and 49 µL dig-300 buffer were added, and the samples were incubated at room temperature for 1 h. Next, the samples were washed twice with 100 µL dig-wash buffer. We subsequently added 200 µL tagmentation buffer and incubated the samples at 37°C for 1 h. The reaction was stopped with 7 µL 0.5 M EDTA, 2 µL 10% SDS, and 1.7 µL 20 mg/mL proteinase K; for single-cell CUT&Tag assays, 5 µL stop buffer was used. After extraction with phenol-chloroform and ethanol precipitation, PCR was performed to amplify the libraries under the following conditions: 72°C for 3 min, 98°C for 30 s, 17 cycles of 98°C for 10 s and 60°C for 5 s, and a final extension at 72°C for 1 min with a hold at 4°C. For single-cell CUT&Tag of human embryos, CUT&Tag was performed in well of a microplate. After tagmentation, single human embryos were transferred to 200µl tubes containing 5% Proteinase K. After embryos lysis, PCR buffer and adaptors were directly added to tubes, normal PCR cycle was performed. Post-PCR clean-up was performed by adding a 1.5× volume of DNA Clean Beads (Vazyme Biotech). Libraries were sequenced on the Illumina NovaSeq 6000 platform according to the manufacturer’s instructions.

**CUT&Tag data analysis.**

We aligned paired-end CUT&Tag reads of H3K18lac to the mm39 reference genome or hg38 human genome using Bowtie2 v2.5.0 software. Unmapped and non-uniquely mapped reads were removed. Next, we pooled the biological replicates for each stage and performed downstream analysis. For peak calling, we converted sequence alignments in BAM format into BEDPE records using the BEDTools function bamtobed. CUT&Tag peaks were called on merged replicates and normalized to input using MACS2 v2.2.7.1 software. Peak annotation was performed using the R package ChIPseeker with the default parameters. Peak comparisons and overlaps were evaluated using the BEDTools suite for autosomal chromosomes. For quantitative analysis, we normalized the read counts by computing the number of RPKMs using merged replicate BAM files with the bamCoverage tool in deepTools software. For the comparison among different samples, we further summed the RPKM values within each 5kb window in the global genome and performed pearson correlation analysis. To minimize batch and cell-type variation, the RPKM values were normalized by Z-score transformation (whole-genome 100 bp bins, excluding outliner regions). Pearson correlation analysis of gene expression levels (FPKM) with normalized RPKM values of H3K18lac signals at promoters was calculated by cor.test() function in R. The washU epigenome browser ([http://epigenomegateway.wustl.edu/browser/](mailto:http://epigenomegateway.wustl.edu/browser/)) was used to visualize H3K18lac CUT&Tag data.

**Statistical analysis.**

Statistical analysis was performed using R v4.2.1 software (R Development Core Team, Vienna, Austria). Data are expressed as means ± standard errors of the mean (SEMs). Differences between means were evaluated using two-tailed Student’s *t*-test. Asterisks indicate significant differences as follows: *P < 0.05, **P < 0.01, and ***P < 0.001.
